# Cardiovascular remodeling: key mechanisms and clinical implications linking sarcopenia and heart failure

**DOI:** 10.3389/fmed.2026.1736824

**Published:** 2026-02-12

**Authors:** Jinghong Yang, Xinyu Zhang, Jun Zhong

**Affiliations:** 1 Department of Orthopedics, Rongchang District People’s Hospital, Chongqing, China; 2 Chongqing Medical University, Chongqing, China; 3 Department of Breast and Thyroid Surgery, The First Affiliated Hospital of Chongqing Medical University, Chongqing, China

**Keywords:** cardiovascular remodeling, chronic inflammation, heart failure, insulin resistance, oxidative stress, sarcopenia

## Abstract

Sarcopenia and heart failure (HF) are interconnected through a bidirectional relationship in which cardiovascular remodeling serves as a critical mediator. This review examines the shared pathophysiological mechanisms—such as chronic inflammation, oxidative stress, and insulin resistance—that underlie both conditions. By integrating recent clinical and experimental evidence, we emphasize the role of cardiovascular remodeling in aggravating muscle loss in sarcopenia and cardiac dysfunction in HF. Furthermore, we discuss novel therapeutic targets directed at disrupting these common pathways. This analysis offers a unified framework for understanding the mechanistic links between sarcopenia and HF, with implications for future research and clinical strategies aimed at enhancing patient outcomes.

## 1 Introduction

Sarcopenia and heart failure (HF) commonly coexist in older adults and are interconnected through a reciprocal interplay that adversely affects clinical morbidity and mortality. Heart failure with reduced ejection fraction (HFrEF) is clinically defined as symptomatic HF with a left ventricular ejection fraction (LVEF) ≤ 40%, as per the 2021 ESC Guidelines and 2022 AHA/ACC/HFSA recommendations. Sarcopenia, as operationalized by the European Working Group on Sarcopenia in Older People (EWGSOP2), is characterized by low muscle strength, reduced muscle quantity or quality, and impaired physical performance. Converging evidence indicates that cardiovascular remodeling acts as a central pathophysiological link between these two conditions (1, 2). While sarcopenia increases susceptibility to HF and accelerates its clinical course, HF itself promotes muscle wasting via mechanisms such as persistent low-grade inflammation, endocrine dysregulation, and physical deconditioning. This bidirectional relationship is particularly pronounced in high-risk phenotypes such as sarcopenic obesity. For example, in diabetic individuals with HF and reduced ejection fraction (HFrEF), the co-occurrence of low muscle mass and obesity correlates with maladaptive left ventricular (LV) remodeling and unfavorable outcomes, underscoring the need for targeted clinical recognition (3, 4).

Cardiovascular remodeling—encompassing structural and functional alterations in the heart and vasculature, including arterial stiffening, LV hypertrophy, and microvascular dysfunction—represents a common pathway in both sarcopenia and HF (5). For the purpose of this review, cardiovascular remodeling is operationally defined as the integrated spectrum of maladaptive structural and functional alterations affecting three interrelated compartments: the myocardium (e.g., hypertrophy, fibrosis), the macrovasculature (e.g., arterial stiffening, endothelial dysfunction), and the microcirculation. These alterations collectively contribute to a state of impaired cardiac efficiency, increased hemodynamic burden, and compromised peripheral perfusion. The mechanistic link to sarcopenia lies in the resultant chronic reduction in oxygen and nutrient delivery to skeletal muscle, concomitant with a systemic milieu of inflammation and metabolic dysregulation that adversely affects muscle protein turnover. A systematic review and meta-analysis support this association, revealing that elevated pulse wave velocity (PWV), a recognized indicator of arterial stiffness, is significantly linked to sarcopenia, implying a shared vascular basis for muscle deterioration and cardiac impairment (6).

Inflammatory and metabolic disturbances further underpin this relationship. Adipokines such as leptin and adiponectin contribute to cardiac remodeling processes; leptin exerts cardiodepressive effects, while adiponectin reflects metabolic dysfunction associated with inflammation and muscle loss in cardiovascular patients (7). Interventions like high-intensity interval training (HIIT) have been shown to improve skeletal muscle perfusion and vascular reactivity in older adults, suggesting that enhancing microcirculatory function and lowering blood pressure may alleviate sarcopenia and its cardiovascular sequelae (8). Emerging strategies, including probiotic supplementation, also exhibit potential in moderating cardiac remodeling and sarcopenia metrics by influencing inflammatory states, oxidative stress, and gut microbiota composition, thereby modulating key pathways such as Wnt signaling involved in muscle homeostasis (5, 9).

A deeper understanding of these interconnected mechanisms is crucial to designing effective interventions that disrupt the vicious cycle linking sarcopenia and HF, with the ultimate goal of improving health outcomes in an aging global population.

## 2 Epidemiological association between sarcopenia and HF

Sarcopenia, characterized by the progressive and generalized loss of skeletal muscle mass, strength, and function, is increasingly recognized as a major comorbidity and a critical determinant of clinical outcomes in heart failure (HF) (2, 10). Epidemiological studies reveal a substantial disease burden, with a global pooled prevalence of approximately 34% among HF patients (5). This prevalence is markedly influenced by clinical setting and disease severity, escalating to 55% in hospitalized cohorts compared to 26% in ambulatory patients, and is further modulated by sex and HF phenotype, being more common in males and those with reduced ejection fraction (HFrEF) (11). The pathophysiological interplay between sarcopenia and HF is driven by a self-perpetuating cycle of chronic systemic inflammation, neurohormonal activation, metabolic dysregulation, and anabolic resistance, which collectively promote muscle protein catabolism. This muscle wasting is not merely a peripheral epiphenomenon but is independently associated with exacerbated physical frailty, diminished functional capacity as measured by tools like handgrip strength, and impaired quality of life (12–14).

Emerging evidence supports a bidirectional predictive relationship between sarcopenia and HF. Sarcopenia, particularly when accompanied by low handgrip strength, independently predicts incident HF in older adults (hazard ratio ≈ 1.5–2.0), likely mediated through vascular dysfunction and chronic inflammation. Conversely, HF—especially HFrEF—predicts accelerated muscle loss attributable to reduced perfusion, anabolic resistance, and physical inactivity. Notably, sarcopenia serves as a strong prognostic indicator, with severe cases conferring a 2.5-fold increased risk of cardiac mortality or HF rehospitalization within 1 year (12, 15, 16). Objective measures such as low phase angle (<5.45°) and reduced spot urinary creatinine concentration have been validated as biomarkers for identifying high-risk patients (17, 18). However, integrated prediction models that combine both sarcopenia and HF remain underdeveloped; future studies should incorporate imaging biomarkers (e.g., pulse-wave velocity, cardiac myosin-binding protein C) along with functional assessments to improve risk stratification. Therefore, early diagnosis and management of sarcopenia—through targeted resistance exercise training and comprehensive nutritional support—represent a promising therapeutic strategy to disrupt this pathophysiological cycle and improve long-term prognosis and wellbeing in patients with HF.

### 2.1 Temporal dynamics and causal interplay: unraveling the sequence

The intricate relationship between cardiovascular remodeling and sarcopenia raises a pivotal question regarding temporal precedence and causal directionality. The sequence is likely bidirectional and phenotype-dependent. In scenarios such as chronic hypertension or primary valvulopathies, maladaptive cardiovascular remodeling may constitute the inciting event. Progressive myocardial fibrosis and vascular stiffening impair cardiac output reserve and peripheral perfusion, ultimately fostering an environment conducive to the development of sarcopenia. Conversely, primary sarcopenia, arising from aging, sedentarism, or nutritional deficiencies, can instigate a pathophysiological cascade characterized by systemic inflammation, insulin resistance, and reduced physical activity. This cascade promotes endothelial dysfunction and arterial stiffening, thereby driving adverse cardiac remodeling and potentially precipitating or exacerbating HF. In clinical practice, particularly among the elderly, these processes frequently coexist and engage in a vicious, self-perpetuating cycle, making the delineation of a primary driver challenging. Future longitudinal studies employing serial, multimodality assessments of cardiac structure/function and muscular parameters are essential to elucidate predominant temporal sequences across distinct clinical phenotypes.

## 3 Shared pathophysiological mechanisms between sarcopenia and heart failure

**FIGURE 1:**
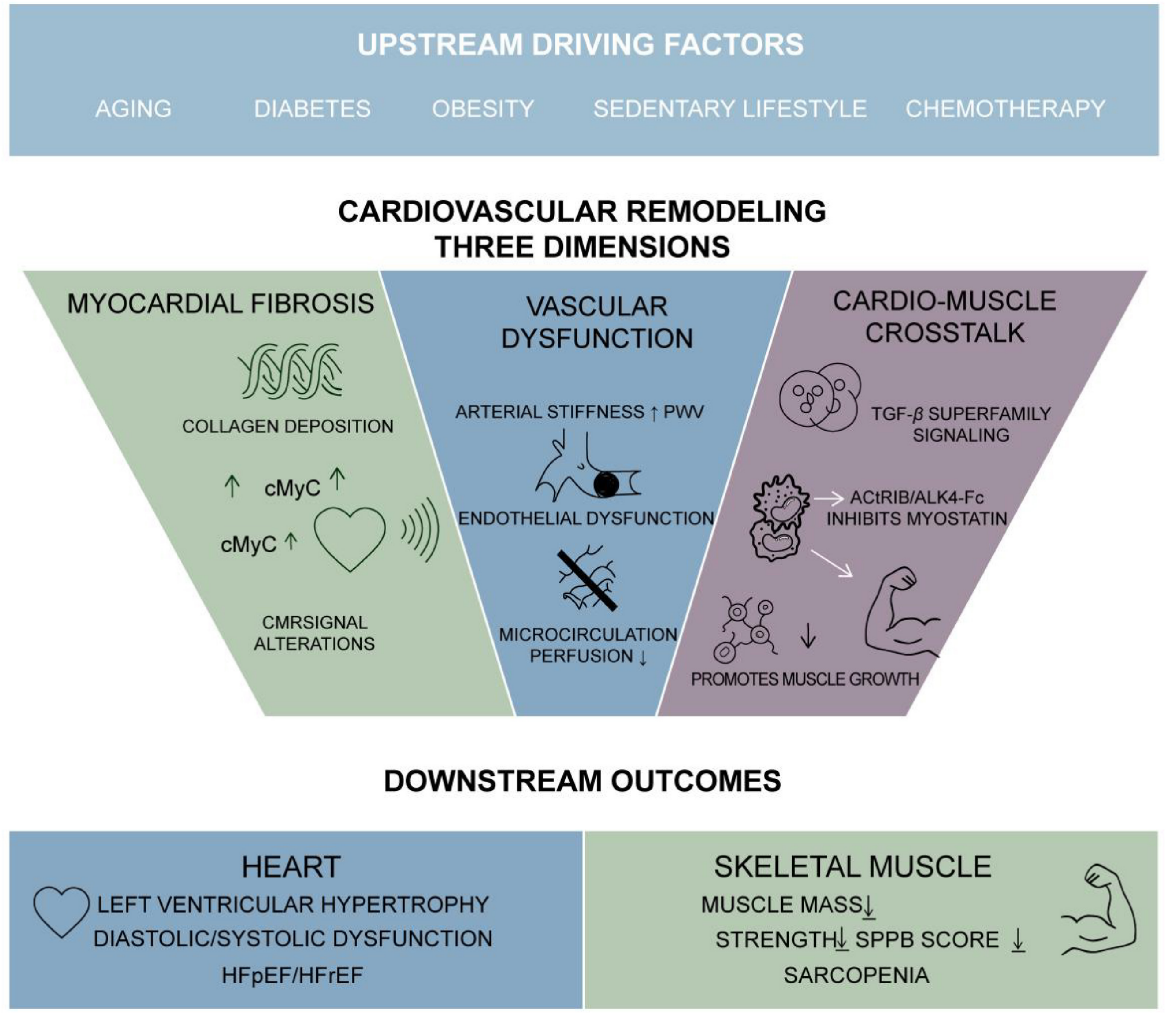
The bidirectional interplay between sarcopenia and heart failure mediated by shared pathophysiological mechanisms.

The interplay between sarcopenia and HF is orchestrated through several overlapping pathways, with chronic inflammation and insulin resistance emerging as central drivers due to their dual impact on cardiac structure and skeletal muscle metabolism (Table 1 and Figure 1).

**TABLE 1:** Shared mechanisms in sarcopenia and heart failure: comparative summary.

Mechanism	Role in sarcopenia	Role in HF	Proposed centrality (high/medium/low)	Rationale
Chronic inflammation	Activates NF-κB → muscle proteolysis	Promotes fibrosis, endothelial dysfunction	High	Directly links muscle wasting and cardiac remodeling
Oxidative stress	Disrupts Ca^2+^ handling, contractile proteins	Induces apoptosis, fibrosis	Medium	Amplifies damage in both tissues
Insulin resistance	↓ PI3K/Akt →↓ protein synthesis	↓ glucose uptake → metabolic shift	High	Core metabolic dysregulation in both
Neurohormonal activation	↑ Catabolism via RAAS and SNS	↑ Preload/afterload, fibrosis	Medium	Modulated by drugs (ACEi/ARB)

### 3.1 The role of chronic inflammation

Chronic inflammation plays a significant role in the pathogenesis of sarcopenia and heart failure (HF). Inflammatory cytokines such as tumor necrosis factor-α (TNF-α) and interleukin-6 (IL-6) are markedly elevated in both conditions (19–21). TNF-α promotes muscle protein degradation by activating the nuclear factor-κB (NF-κB) pathway, thereby contributing to the development of sarcopenia (19, 22, 23). Concurrently, TNF-α exacerbates the pathological progression of heart failure by inhibiting myocardial contractility (24). IL-6 further intensifies cardiac remodeling by promoting myocardial fibrosis and vascular endothelial dysfunction. Studies indicate that serum IL-6 levels in HF patients correlate positively with the severity of left ventricular dysfunction, while IL-6 levels in sarcopenia patients are closely associated with diminished muscle mass and function (25–27). The combined action of these inflammatory mediators not only accelerates functional decline in both muscle and heart but also amplifies the inflammatory response by activating downstream signaling pathways (such as JAK/STAT), thereby establishing a vicious cycle (28).

The inflammatory response also plays a central role in cardiovascular remodeling. Under chronic inflammatory conditions, inflammatory mediators such as TNF-α and IL-6 promote myocardial fibrosis and vascular endothelial dysfunction, leading to left ventricular hypertrophy and diastolic dysfunction (29). Furthermore, inflammation accelerates extracellular matrix degradation by activating matrix metalloproteinases (MMPs), thereby exacerbating cardiac structural alterations. In patients with sarcopenia, the persistent release of inflammatory mediators not only causes muscle wasting but also indirectly promotes cardiovascular remodeling by impairing myocardial cell energy metabolism and contractile function (21, 30, 31). For instance, TNF-α exacerbates cardiac dysfunction by suppressing insulin signaling pathways and intensifying insulin resistance in cardiomyocytes. Consequently, inflammation serves not only as a shared pathological basis for sarcopenia and HF but also as a crucial bridge linking the two conditions.

### 3.2 The contribution of oxidative stress

Oxidative stress plays a critical role in the pathogenesis of both sarcopenia and HF. Reactive oxygen species (ROS) accumulate due to mitochondrial dysfunction and impaired antioxidant defenses, leading to cellular damage (32, 33). In skeletal muscle, excessive ROS disrupts calcium handling and contractile proteins, accelerating muscle atrophy. In the myocardium, ROS contribute to cardiomyocyte apoptosis, fibrosis, and impaired excitation-contraction coupling (34). Oxidative stress also exacerbates insulin resistance, further impairing muscle and cardiac metabolism. Therapeutic strategies targeting ROS, such as antioxidants or mitochondrial enhancers, may help preserve muscle mass and cardiac function in these conditions (35–37).

Beyond this, oxidative stress exerts strikingly similar effects on both cardiac and skeletal muscle function. Within cardiomyocytes, reactive oxygen species (ROS) impair mitochondrial DNA and membrane lipids, leading to reduced ATP production and abnormal calcium regulation, which in turn disrupts cardiac contraction and relaxation. Similarly, in skeletal muscle, ROS inhibit myofibril synthesis while promoting their degradation, resulting in diminished muscle mass and strength (38). Notably, oxidative stress further exacerbates functional decline by impairing vascular endothelial function, thereby reducing blood perfusion to both skeletal and cardiac muscle. For instance, impaired skeletal muscle microvascular function in HF patients correlates closely with elevated ROS levels, while antioxidant therapies (such as N-acetylcysteine) can partially reverse this damage (39).

### 3.3 Insulin resistance and metabolic dysregulation

Insulin resistance is a common metabolic feature of sarcopenia and heart failure. In sarcopenia, insulin resistance reduces muscle protein synthesis by inhibiting the PI3K/Akt signaling pathway, whilst activating the UPS system to accelerate protein degradation (40). In HF, insulin resistance diminishes glucose uptake by cardiomyocytes, shifting energy dependence toward fatty acid oxidation (41). This process is not only inefficient but also generates substantial ROS, further damaging myocardial tissue. Furthermore, insulin resistance exacerbates inflammatory responses in muscle and heart tissue by promoting the release of inflammatory mediators such as TNF-α and IL-6. Clinical studies demonstrate a significant correlation between the insulin resistance index (HOMA-IR) in HF patients and the severity of sarcopenia, indicating a close metabolic link between the two conditions (42).

Metabolic abnormalities (such as hyperglycemia and dyslipidemia) similarly promote cardiovascular remodeling through multiple pathways. Hyperglycemia activates inflammatory and oxidative stress pathways via advanced glycation end products (AGEs), leading to myocardial fibrosis and vascular stiffening (43). Dyslipidemia exacerbates cardiac contractile dysfunction by promoting lipotoxicity and impairing mitochondrial function in cardiomyocytes (44). In patients with sarcopenia, metabolic abnormalities further deteriorate muscle function by reducing blood flow perfusion and energy supply to muscles. For instance, skeletal muscle microvascular dysfunction in diabetic patients is closely associated with metabolic abnormalities, and improved metabolic control (e.g., via SGLT2 inhibitors) can partially reverse this damage. Consequently, metabolic abnormalities represent not only a shared pathophysiological basis for sarcopenia and HF but also a critical target for intervention (45, 46).

## 4 The key role of cardiovascular remodeling

**FIGURE 2:**
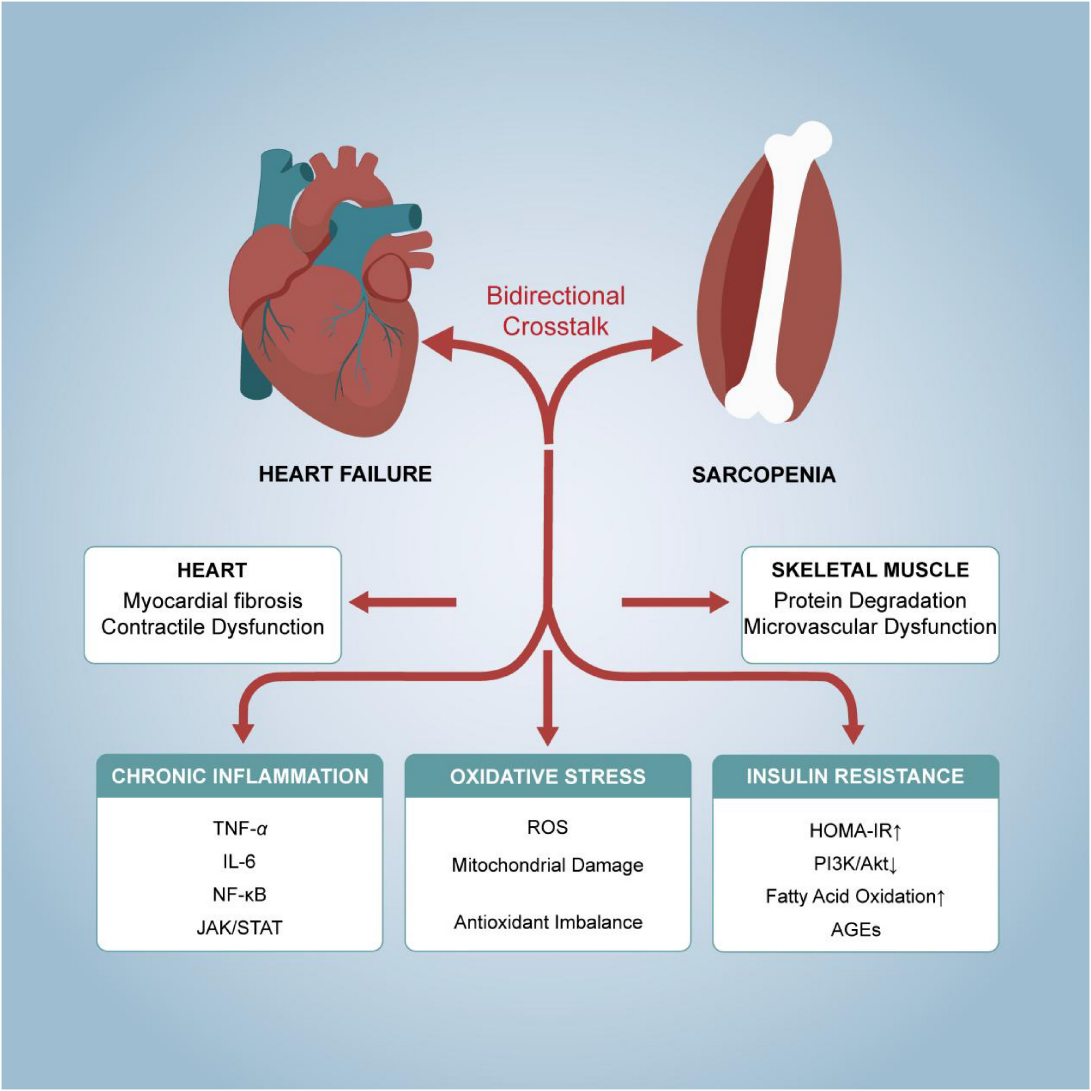
Cardiovascular remodeling as a central mediator linking sarcopenia and heart failure.

Cardiovascular remodeling represents a fundamental pathological process in numerous cardiac conditions, characterized by structural and functional alterations in both the myocardium and vasculature (Figure 2). This complex process involves myocardial fibrosis, vascular dysfunction, and intricate cross-talk between cardiac and skeletal muscle systems. In salt-sensitive cardiovascular disease, high dietary sodium promotes myocardial fibrosis through non-hemodynamic mechanisms involving redox-sensitive and profibrotic pathways, contributing to diastolic dysfunction and potentially heart failure with preserved ejection fraction (HFpEF) (47, 48). Cardiac magnetic resonance (CMR) imaging has emerged as a crucial diagnostic tool for assessing myocardial fibrosis, ventricular remodeling, and mitral regurgitation severity, enabling early detection before irreversible damage occurs (49, 50). The development of novel biomarkers such as cardiac myosin binding protein C (cMyC) further enhances our understanding of chronic myocardial injury and left ventricular remodeling in general populations. Chemotherapy-induced cardiovascular injury demonstrates how myocardial edema, inflammation, and fibrosis can be detected through advanced imaging techniques, highlighting the sensitivity of CMR in identifying early, potentially reversible tissue remodeling. Machine learning approaches have improved risk stratification in mitral valve prolapse by identifying distinct echocardiographic phenotypes associated with myocardial fibrosis and clinical outcomes, emphasizing the importance of integrating left ventricular dilatation and fibrosis assessment with quantitative mitral regurgitation evaluation for optimal intervention timing (51, 52).

### 4.1 Myocardial fibrosis and sarcopenia

Myocardial fibrosis and sarcopenia share common pathophysiological pathways that extend beyond their individual clinical manifestations, representing interconnected processes in cardiovascular and musculoskeletal health (1). Myocardial fibrosis, characterized by fibroblast activation and excessive extracellular matrix collagen deposition, contributes significantly to ventricular stiffness and both systolic and diastolic dysfunction, ultimately progressing to heart failure (53). The relationship between cardiac fibrosis and muscle wasting becomes evident through several mechanisms, including shared inflammatory pathways, neurohormonal activation, and metabolic dysregulation (54). In primary mitral regurgitation, substantial left ventricular volume overload leads to adverse cardiac remodeling and myocardial replacement fibrosis, which parallels the muscle degradation processes observed in sarcopenia. The emerging application of sodium magnetic resonance imaging (23Na-MRI) provides novel insights into myocardial sodium overload and its association with fibrotic remodeling, while circulating biomarkers like cMyC demonstrate associations with focal myocardial fibrosis and left ventricular remodeling in general populations (55–57). These imaging and biomarker advancements reveal how cardiac structural changes may reflect or contribute to systemic muscle wasting processes. Furthermore, therapeutic interventions targeting myocardial fibrosis, including mineralocorticoid receptor antagonists, angiotensin receptor blockers, and sodium-glucose cotransport 2 inhibitors, may have implications for addressing skeletal muscle deterioration, suggesting potential cross-benefits in managing both cardiac and musculoskeletal health.

### 4.2 Vascular dysfunction and skeletal muscle atrophy

Vascular dysfunction and skeletal muscle atrophy maintain a bidirectional relationship where impaired vascular function contributes to muscle wasting, while muscle deterioration further exacerbates vascular compromise. The activin receptor signaling pathway provides crucial insights into this interconnection, as demonstrated by the heterodimeric ligand-trapping fusion protein ActRIIB:ALK4-Fc, which exhibits distinct ligand binding properties affecting both vascular regulation and muscle growth (58). This therapeutic agent specifically sequesters ActRIIB ligands known to inhibit muscle growth while avoiding the trapping of vascular regulatory ligand bone morphogenetic protein 9 (BMP9), highlighting the delicate balance required in modulating these interconnected systems. The differential effects observed in retinal explant assays between ActRIIB:ALK4-Fc and its homodimeric variant ActRIIB-Fc underscore the complexity of vascular-muscle interactions. In clinical contexts such as chemotherapy-induced cardiovascular injury, vascular remodeling occurs alongside myocardial tissue changes, with CMR capable of detecting not only established morphologic and functional abnormalities but also early signs of vascular compromise. The improvement in neuromuscular junction abnormalities and alleviation of acute muscle fibrosis observed in murine models of Duchenne muscular dystrophy and amyotrophic lateral sclerosis following ActRIIB:ALK4-Fc treatment further emphasizes the therapeutic potential of targeting shared pathways between vascular function and muscle preservation (59).

### 4.3 Cardiac-skeletal muscle cross-talk

The pathophysiological interplay between heart failure (HF) and sarcopenia is mediated by specific molecular pathways, with the TGF-β/activin signaling axis being a central mechanism (60). Elevated circulating activins in HF activate the ActRIIB/ALK4 receptors on skeletal muscle, directly promoting proteolysis and muscle wasting. This explains the efficacy of agents like ActRIIB:ALK4-Fc in preclinical models, which simultaneously ameliorate cardiac dysfunction and muscle loss by blocking this shared pathway (61).

Clinically, this organ crosstalk is substantiated by biomarkers and phenotypic clustering. For instance, cardiac-specific biomarkers such as cMyC correlate not only with myocardial injury but also with systemic muscle depletion. Similarly, machine learning-derived echocardiographic phenotypes link specific patterns of cardiac remodeling to a higher risk of generalized sarcopenia, indicating that primary cardiac pathology can broadcast systemic signals affecting muscle health (59). Furthermore, circulating biomarkers like cMyC reflect not only cardiac remodeling and focal myocardial fibrosis but also associate with cardiovascular risk factors and ventricular dysfunction, serving as potential indicators of systemic muscle health status (59).

Future research should move beyond documenting coexistence to defining the precise hierarchy and timing of signals within this cross-talk. Key questions include whether sarcopenia in HF is primarily driven by specific cardiac-derived factors (e.g., from failing myocardium versus activated fibroblasts) or by amplified systemic inflammation. Furthermore, the role of skeletal muscle as an endocrine organ, potentially releasing myokines that feedback to accelerate or attenuate cardiac remodeling, remains poorly explored. Defining these directional signals will be crucial for identifying whether therapeutic strategies should primarily target the heart to protect muscle, the muscle to support the heart, or nodal points within their shared signaling network.

## 5 Clinical research evidence on sarcopenia and heart failure

### 5.1 Recent meta-analysis summarizing the association between sarcopenia and HF

Recent meta-analyses have revealed a strong association between sarcopenia and heart failure (HF). A systematic review and meta-analysis involving 5,476 participants demonstrated that pulse wave velocity was significantly higher in individuals with sarcopenia compared to those without (SMD = 0.73, 95% CI 0.39–1.08), suggesting a marked association between increased aortic stiffness and sarcopenia (62, 63). The study further found that elevated PWV positively correlates with the risk of developing sarcopenia, suggesting vascular dysfunction may represent one potential mechanism underlying the co-morbidity of sarcopenia and HF. Another cardiac MRI study in diabetic patients with HFrEF demonstrated that sarcopenic obesity (SO) patients exhibited more severe left ventricular dilatation and dysfunction, with a 3-fold increased risk of cardiovascular adverse events compared to non-SO patients (HR = 3.03, 95% CI 1.39–6.63) (64). Collectively, these findings suggest that sarcopenia not only constitutes an independent risk factor for HF but may also worsen HF prognosis by influencing cardiac remodeling and vascular dysfunction.

### 5.2 Interventional studies: effects of exercise training and nutritional supplementation

Regarding intervention measures, high-intensity interval training has demonstrated dual improvements for both sarcopenia and heart failure. A randomized controlled trial found that after 6 weeks of HIIT, skeletal muscle microvascular reactivity to contraction significantly increased in older adults aged 65–85 years (blood perfusion rising from 1.8 ± 0.63 to 2.3 ± 0.8 AU), alongside concurrent improvements in cardiorespiratory fitness and blood pressure control (65, 66). In addition, regarding nutritional interventions, probiotic supplementation offers multiple benefits under cardiac remodeling conditions: by modulating the Wnt signaling pathway (significantly correlated with improved grip strength, *p* < 0.05), and reducing inflammatory markers such as hs-CRP, it may concurrently mitigate sarcopenia and HF progression. Notably, improvements in Short Physical Performance Battery (SPPB) scores were closely correlated with changes in muscle metabolic markers including Dkk-3 and SREBP-1, suggesting that combined interventions may produce synergistic effects via the metabolic-inflammatory axis (67).

### 5.3 Biomarkers for predicting sarcopenia and HF risk

Biomarker studies have revealed the pivotal role of leptin and adiponectin in the association between sarcopenia and heart failure (56). Studies in cardiovascular surgery patients demonstrate that serum adiponectin levels correlate positively with cardiac function parameters such as BNP and left atrial diameter (LAD), whilst leptin correlates positively with LVEF but negatively with LV mass index (68, 69). Notably, adiponectin independently predicts sarcopenia in male patients (OR not reported, *p* < 0.05), and the possible mechanisms potentially involve activation of the pro - inflammatory factor TNF - α and abnormal muscle protein metabolism (7). Additionally, skeletal muscle index (SMI) of the thoracic skeleton—as a surrogate marker for muscle mass—effectively distinguishes high - risk phenotypes in HFrEF patients (70). When SMI < 42.75 cm^2^/m^2^ coexists with obesity, patients exhibit more pronounced left ventricular remodeling and poorer outcomes. These biomarkers provide objective evidence for early identification of high - risk populations for sarcopenia - associated HF (71). We have summarized some potential biomarkers and their correlations and shortcomings, which are summarized in Table 2.

**TABLE 2:** Candidate biomarkers linking sarcopenia and heart failure.

Biomarker	Sample type	Primary clinical context	Role/association	Key limitations
Leptin	Serum/plasma	Obesity, HF, cachexia	Positively correlates with LVEF; negatively with LV mass index. Reflects adiposity and may indicate adipose tissue dysfunction.	Strongly influenced by total fat mass; exhibits complex, non-linear relationships with clinical outcomes.
Adiponectin	Serum/plasma	HF, metabolic syndrome, sarcopenia	Elevated levels predict sarcopenia in males; correlates with BNP and left atrial diameter. A marker of metabolic dysregulation.	Paradoxically high in advanced HF/cachexia (“adiponectin paradox”); lacks disease specificity.
Thoracic skeletal muscle index (SMI)	CT/MRI	HFrEF, sarcopenic obesity	Values < 42.75 cm^2^/m^2^, especially with obesity, identify adverse LV remodeling and poor prognosis. Direct quantitative measure of muscle mass.	Requires advanced imaging (cost, radiation exposure for CT); diagnostic thresholds may vary across populations.
Phase angle	Bioelectrical impedance analysis (BIA)	HF, malnutrition, sarcopenia	A value < 5.45° is indicative of risk; reflects cellular membrane integrity and body cell mass.	Sensitive to hydration status; results vary with measurement device and protocol.
Cardiac myosin-binding protein C (cMyC)	Serum	General population, chronic myocardial injury	Associated with focal myocardial fibrosis and left ventricular remodeling; a potential indicator of systemic musculoskeletal health.	Still in exploratory research phases; clinical utility within the sarcopenia-HF dyad awaits validation.
Pulse wave velocity (PWV)	Non-invasive vascular assessment	Vascular aging, sarcopenia, HF	Significantly elevated in individuals with sarcopenia; provides a direct link between arterial stiffness and muscle loss.	Requires specialized equipment; measurements are influenced by concurrent blood pressure.

## 6 Future research directions and clinical implications

### 6.1 Targeting common mechanisms for therapeutic intervention

Future research should prioritize the development of therapies targeting the shared pathological mechanisms between HF and sarcopenia. For instance, artificial intelligence (AI) holds promise for these specific conditions by analyzing multi-omics data to identify novel therapeutic targets, predict individual patient responses, and optimize treatment strategies, thereby moving toward precision medicine in cardiometabolic-musculoskeletal disorders (72, 73). Another relevant approach is the exploration of bioactive compounds, such as resveratrol, for their multi-dimensional benefits including anti-inflammatory and mitochondrial-protective effects, which are pertinent to both cardiac and muscle pathology. However, their clinical application is limited by poor bioavailability, necessitating research into improved formulations or delivery systems for these patient populations.

### 6.2 Multidisciplinary integrated intervention strategies

**FIGURE 3:**
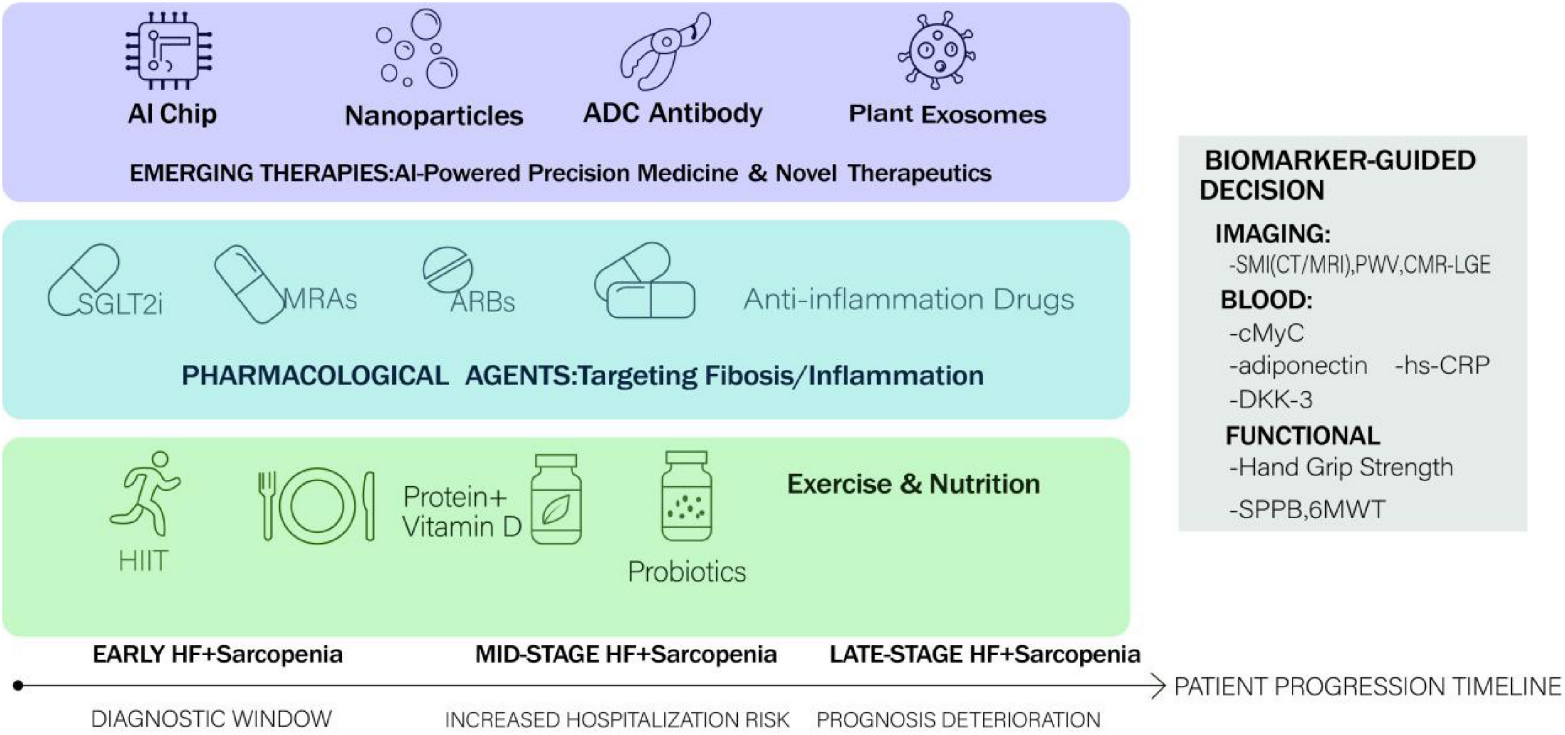
Integrated and personalized therapeutic strategies for disrupting the sarcopenia-heart failure cycle.

A multidisciplinary approach is essential for managing the sarcopenia-heart failure continuum, integrating nutritional support, pharmacotherapy, and tailored physical interventions (Figure 3). For HF, this includes guideline-directed medical therapy (e.g., ACE inhibitors, beta-blockers), salt-restricted diets, and cardiac rehabilitation. For sarcopenia, management combines protein and vitamin D supplementation with resistance and aerobic training to improve muscle mass and function (74). AI can aid in personalizing these strategies through the integrated analysis of body composition, cardiac function parameters, and biomarker profiles. Incorporating psychosocial support is also crucial for enhancing adherence and quality of life. Future initiatives should focus on standardizing multidisciplinary care pathways and utilizing digital health tools to improve coordination between cardiology, geriatrics, nutrition, and rehabilitation services (75).

### 6.3 Development of personalized treatment approaches

Personalized medicine for HF and sarcopenia involves tailoring interventions based on individual patient profiles, leveraging biomarkers, AI, and advanced delivery systems. AI algorithms analyzing patient-specific multi-omics data can help predict disease progression, the risk of cachexia/sarcopenia, or response to specific therapies, enabling timely intervention. Furthermore, technologies like nanoparticle-based delivery systems could be adapted to enhance the bioavailability and targeted action of drugs or nutraceuticals relevant to muscle and cardiac health (e.g., specific anti-inflammatory agents). Plant-derived extracellular vesicles (EVs) are also being explored as novel delivery platforms with potential applications in regenerative medicine, which may be relevant for tissue repair in these conditions. The primary focus for future research must be the validation of such personalized approaches in large-scale clinical trials specific to HF and sarcopenia, incorporating robust functional and patient-reported outcomes.

### 6.4 Current guideline incorporation of sarcopenia in HF management

A review of major HF guidelines reveals limited explicit integration of sarcopenia. The 2022 AHA/ACC/HFSA guidelines mention “muscle wasting” as a comorbidity but lack screening recommendations. The 2021 ESC Guidelines note nutritional support in cachexia but do not address sarcopenia specifically. Chinese HF guidelines (2024) acknowledge sarcopenia as a prognostic factor but offer no standardized assessment protocol. This gap underscores the need for guideline updates that incorporate routine sarcopenia screening (e.g., SARC-F questionnaire, grip strength) in HF clinics, along with tailored interventions (resistance exercise, protein supplementation) to break the detrimental cycle.

## 7 Conclusion

In conclusion, cardiovascular remodeling emerges as a pivotal pathophysiological nexus linking sarcopenia and heart failure, driven by shared mechanisms of chronic inflammation, oxidative stress, and metabolic dysregulation. This interplay establishes a vicious cycle wherein cardiac dysfunction and skeletal muscle wasting mutually reinforce each other, leading to worsened clinical outcomes. Evidence underscores the prognostic significance of sarcopenia in HF populations and highlights the potential of targeting common pathways—such as with exercise training, nutritional support, and novel pharmacological agents—to disrupt this detrimental crosstalk. Moving forward, future research should prioritize the development of integrated, multidisciplinary management strategies and leverage advances in biomarker discovery, imaging technologies, and artificial intelligence to enable early detection and personalized therapeutic interventions. By addressing both cardiac and muscular health in a coordinated manner, we can aspire to improve functional capacity, quality of life, and long-term survival for patients afflicted with these interrelated conditions.

## References

[1] Curcio F Testa G Liguori I Papillo M Flocco V Panicara V Sarcopenia and heart failure. *Nutrients*. (2020) 12:211. 10.3390/nu12010211 31947528 PMC7019352

[2] Lena A Anker MS Springer J. Muscle wasting and sarcopenia in heart failure-the current state of science. *Int J Mol Sci*. (2020) 21:6549. 10.3390/ijms21186549 32911600 PMC7555939

[3] Campbell P Rutten FH Lee MM Hawkins NM Petrie MC. Heart failure with preserved ejection fraction: everything the clinician needs to know. *Lancet*. (2024) 403:1083–92. 10.1016/S0140-6736(23)02756-3 38367642

[4] Redfield MM Borlaug BA. Heart failure with preserved ejection fraction: a review. *JAMA*. (2023) 329:827–38. 10.1001/jama.2023.2020 36917048

[5] de Jorge-Huerta L Marco-Alacid C Grande C Velardo Andrés CA. Narrative review of the diagnosis and treatment of Sarcopenia and malnutrition in patients with heart failure. *Nutrients*. (2024) 16:2717. 10.3390/nu16162717 39203852 PMC11357594

[6] Amarasekera AT Chang D Schwarz P Tan TC. Does vascular endothelial dysfunction play a role in physical frailty and sarcopenia? A systematic review. *Age Ageing*. (2021) 50:725–32. 10.1093/ageing/afaa237 33951149

[7] Komici K Dello Iacono A De Luca A Perrotta F Bencivenga L Rengo G Adiponectin and Sarcopenia: a systematic review with meta-analysis. *Front Endocrinol*. (2021) 12:576619. 10.3389/fendo.2021.576619 33935962 PMC8082154

[8] Liu QQ Xie WQ Luo YX Li YD Huang WH Wu YX High intensity interval training: a potential method for treating Sarcopenia. *Clin Interv Aging*. (2022) 17:857–72. 10.2147/CIA.S366245 35656091 PMC9152764

[9] Fernández-Pombo A Rodríguez-Carnero G Castro AI Cantón-Blanco A Seoane LM Casanueva FF Relevance of nutritional assessment and treatment to counteract cardiac cachexia and Sarcopenia in chronic heart failure. *Clin Nutr*. (2021) 40:5141–55. 10.1016/j.clnu.2021.07.027 34461588

[10] Krysztofiak H Wleklik M Migaj J Dudek M Uchmanowicz I Lisiak M Cardiac cachexia: a well-known but challenging complication of heart failure. *Clin Interv Aging*. (2020) 15:2041–51. 10.2147/CIA.S273967 33173285 PMC7646468

[11] Honda S Uemura Y Shibata R Sekino T Takemoto K Ishikawa S Clinical implications of severe sarcopenia in Japanese patients with acute heart failure. *Geriatr Gerontol Int*. (2022) 22:477–82. 10.1111/ggi.14389 35460315

[12] Prokopidis K Irlik K Hendel M Piaśnik J Lip GYH Nabrdalik K. Prognostic impact and prevalence of cachexia in patients with heart failure: a systematic review and meta-analysis. *J Cachexia Sarcopenia Muscle*. (2024) 15:2536–43. 10.1002/jcsm.13596 39478303 PMC11634528

[13] Zhang Y Zhang J Ni W Yuan X Zhang H Li P Sarcopenia in heart failure: a systematic review and meta-analysis. *ESC Heart Fail*. (2021) 8:1007–17. 10.1002/ehf2.13255 33576177 PMC8006658

[14] Campos PIC Diz JBM Leopoldino AAO Malachias MVB. Heart failure in patients with Sarcopenia: systematic review and meta-analysis. *Ann Geriatr Med Res*. (2025) 29:295–304. 10.4235/agmr.24.0186 40205832 PMC12489607

[15] Liu Y Su M Lei Y Tian J Zhang L Xu D. Sarcopenia predicts adverse prognosis in patients with heart failure: a systematic review and meta-analysis. *Rev Cardiovasc Med*. (2023) 24:273. 10.31083/j.rcm2409273 39076387 PMC11270102

[16] Yang TR Ji P Deng X Feng XX He ML Wang RR Ct-based diagnosis of sarcopenia as a prognostic factor for postoperative mortality after elective open-heart surgery in older patients: a cohort-based systematic review and meta-analysis. *Front Public Health*. (2024) 12:1378462. 10.3389/fpubh.2024.1378462 39040869 PMC11261807

[17] Bernal-Ceballos F Castillo-Martínez L Reyes-Paz Y Villanueva-Juárez JL Hernández-Gilsoul T. Clinical application of phase angle and BIVA Z-score analyses in patients admitted to an emergency department with acute heart failure. *J Vis Exp.* (2023) e65660. 10.3791/65660 37458449

[18] Malinowska-Borowska J Kulik A Buczkowska M Ostręga W Stefaniak A Piecuch M Nutritional and non-nutritional predictors of low spot urinary creatinine concentration in patients with heart failure. *Nutrients*. (2021) 13:3994. 10.3390/nu13113994 34836249 PMC8619433

[19] Luo Q Zhang Q Kong Y Wang S Wei Q. Heart failure, inflammation and exercise. *Int J Biol Sci*. (2025) 21:3324–50. 10.7150/ijbs.109917 40520009 PMC12160080

[20] Livshits G Kalinkovich A. Restoration of epigenetic impairment in the skeletal muscle and chronic inflammation resolution as a therapeutic approach in sarcopenia. *Ageing Res Rev*. (2024) 96:102267. 10.1016/j.arr.2024.102267 38462046

[21] Bian AL Hu HY Rong YD Wang J Wang JX Zhou XZ. A study on relationship between elderly sarcopenia and inflammatory factors IL-6 and TNF-α. *Eur J Med Res*. (2017) 22:25. 10.1186/s40001-017-0266-9 28701179 PMC5508730

[22] Ueland T Yndestad A Dahl CP Gullestad L Aukrust P. TNF revisited: osteoprotegerin and TNF-related molecules in heart failure. *Curr Heart Fail Rep*. (2012) 9:92–100. 10.1007/s11897-012-0088-6 22453763

[23] Wu Q Zou Q Long S Deng H Cui Y. METTL3 promotes oxidative stress and inflammation in myoblasts during chronic kidney disease related sarcopenia through the TLR4 NF-κB pathway. *Microbiol Immunol*. (2025) 69:608–18. 10.1111/1348-0421.70014 41077943

[24] Francis GS. TNF-alpha and heart failure. The difference between proof of principle and hypothesis testing. *Circulation*. (1999) 99:3213–4. 10.1161/01.cir.99.25.3213 10385490

[25] Alogna A Koepp KE Sabbah M Espindola Netto JM Jensen MD Kirkland JL Interleukin-6 in patients with heart failure and preserved ejection fraction. *JACC Heart Fail*. (2023) 11:1549–61. 10.1016/j.jchf.2023.06.031 37565977 PMC10895473

[26] Ridker PM Rane M. Interleukin-6 signaling and anti-interleukin-6 therapeutics in cardiovascular disease. *Circ Res*. (2021) 128:1728–46. 10.1161/CIRCRESAHA.121.319077 33998272

[27] Berger M März W Niessner A Delgado G Kleber M Scharnagl H IL-6 and hsCRP predict cardiovascular mortality in patients with heart failure with preserved ejection fraction. *ESC Heart Fail*. (2024) 11:3607–15. 10.1002/ehf2.14959 39003598 PMC11631318

[28] Hu C Zhang X Zhang N Wei WY Li LL Ma ZG Osteocrin attenuates inflammation, oxidative stress, apoptosis, and cardiac dysfunction in doxorubicin-induced cardiotoxicity. *Clin Transl Med*. (2020) 10:e124. 10.1002/ctm2.124 32618439 PMC7418805

[29] Altamura M D’Andrea G Angelini E Tortorelli FMP Balzotti A Porcelli P Psychosomatic syndromes are associated with IL-6 pro-inflammatory cytokine in heart failure patients. *PLoS One*. (2022) 17:e0265282. 10.1371/journal.pone.0265282 35271674 PMC8912235

[30] Bano G Trevisan C Carraro S Solmi M Luchini C Stubbs B Inflammation and sarcopenia: a systematic review and meta-analysis. *Maturitas*. (2017) 96:10–5. 10.1016/j.maturitas.2016.11.006 28041587

[31] Pan L Xie W Fu X Lu W Jin H Lai J Inflammation and sarcopenia: a focus on circulating inflammatory cytokines. *Exp Gerontol*. (2021) 154:111544. 10.1016/j.exger.2021.111544 34478826

[32] Cao J Zhai Y Li K Li J Tian X Zhang J ROS accelerates the progression of hypertrophic cardiomyopathy. *Genes Dis*. (2026) 13:101741. 10.1016/j.gendis.2025.101741 41158753 PMC12555768

[33] Xu S Ilyas I Little PJ Li H Kamato D Zheng X Endothelial dysfunction in atherosclerotic cardiovascular diseases and beyond: from mechanism to pharmacotherapies. *Pharmacol Rev*. (2021) 73:924–67. 10.1124/pharmrev.120.000096 34088867

[34] Peng F Liao M Jin W Liu W Li Z Fan Z 2-APQC, a small-molecule activator of Sirtuin-3 (SIRT3), alleviates myocardial hypertrophy and fibrosis by regulating mitochondrial homeostasis. *Signal Transduct Target Ther*. (2024) 9:133. 10.1038/s41392-024-01816-1 38744811 PMC11094072

[35] Blokhina O Virolainen E Fagerstedt KV. Antioxidants, oxidative damage and oxygen deprivation stress: a review. *Ann Bot.* (2003) 91:179–94. 10.1093/aob/mcf118 12509339 PMC4244988

[36] Halliwell B. Understanding mechanisms of antioxidant action in health and disease. *Nat Rev Mol Cell Biol*. (2024) 25:13–33. 10.1038/s41580-023-00645-4 37714962

[37] Zhang X Qu H Yang T Liu Q Zhou H. Astragaloside IV attenuate MI-induced myocardial fibrosis and cardiac remodeling by inhibiting ROS/caspase-1/GSDMD signaling pathway. *Cell Cycle*. (2022) 21:2309–22. 10.1080/15384101.2022.2093598 35770948 PMC9586672

[38] Kyrychenko V Poláková E Janíček R Shirokova N. Mitochondrial dysfunctions during progression of dystrophic cardiomyopathy. *Cell Calcium*. (2015) 58:186–95. 10.1016/j.ceca.2015.04.006 25975620 PMC4501876

[39] Souza ACDAH Troschel AS Marquardt JP Hadžić I Foldyna B Moura FA Skeletal muscle adiposity, coronary microvascular dysfunction, and adverse cardiovascular outcomes. *Eur Heart J*. (2025) 46:1112–23. 10.1093/eurheartj/ehae827 39827905 PMC13376131

[40] Gellhaus B Böker KO Schilling AF Saul D. Therapeutic consequences of targeting the IGF-1/PI3K/AKT/FOXO3 axis in sarcopenia: a narrative review. *Cells*. (2023) 12:2787. 10.3390/cells12242787 38132107 PMC10741475

[41] Erqou S Adler AI Challa AA Fonarow GC Echouffo-Tcheugui JB. Insulin resistance and incident heart failure: a meta-analysis. *Eur J Heart Fail*. (2022) 24:1139–41. 10.1002/ejhf.2531 35502564 PMC9262840

[42] Castillo Costa Y Mauro V Fairman E Charask A Olguín L Cáceres L Prognostic value of insulin resistance assessed by HOMA-IR in non-diabetic patients with decompensated heart failure. *Curr Probl Cardiol*. (2023) 48:101112. 10.1016/j.cpcardiol.2022.101112 35007641

[43] Palmer AK Xu M Zhu Y Pirtskhalava T Weivoda MM Hachfeld CM Targeting senescent cells alleviates obesity-induced metabolic dysfunction. *Aging Cell*. (2019) 18:e12950. 10.1111/acel.12950 30907060 PMC6516193

[44] Hu Q Zhang H Gutiérrez Cortés N Wu D Wang P Zhang J Increased Drp1 acetylation by lipid overload induces cardiomyocyte death and heart dysfunction. *Circ Res*. (2020) 126:456–70. 10.1161/CIRCRESAHA.119.315252 31896304 PMC7035202

[45] Jia G Hill MA Sowers JR. Diabetic cardiomyopathy: an update of mechanisms contributing to this clinical entity. *Circ Res*. (2018) 122:624–38. 10.1161/CIRCRESAHA.117.311586 29449364 PMC5819359

[46] Fang Z Raza U Song J Lu J Yao S Liu X Systemic aging fuels heart failure: molecular mechanisms and therapeutic avenues. *ESC Heart Fail*. (2025) 12:1059–80. 10.1002/ehf2.14947 39034866 PMC11911610

[47] Wang XH Yang JY Lei ZW Zheng XT Xie HY Huang H SIRT3 activation by SGLT2 inhibitor mitigates endothelial-to-mesenchymal transition in dahl salt-sensitive rats induced by high-salt diet. *Am J Hypertens.* (2025) [Online ahead of print.]. 10.1093/ajh/hpaf209. 41148059

[48] Lang H Li Q Yu H Li P Lu Z Xiong S Activation of TRPV1 attenuates high salt-induced cardiac hypertrophy through improvement of mitochondrial function. *Br J Pharmacol*. (2015) 172:5548–58. 10.1111/bph.12987 25339153 PMC4667858

[49] Vratonjic J Jovanovic I Petrovic O Paunovic I Boricic-Kostic M Tesic M Multimodality imaging for the management of patients with primary mitral regurgitation. *J Clin Ultrasound*. (2022) 50:1051–9. 10.1002/jcu.23335 36218209

[50] Axel L Phan TS Metaxas DN. Visualization and analysis of multidimensional cardiovascular magnetic resonance imaging: challenges and opportunities. *Front Cardiovasc Med*. (2022) 9:919810. 10.3389/fcvm.2022.919810 35859582 PMC9289269

[51] Huttin O Le Tourneau T Filippetti L Pace N Sellal JM Beaumont M A new evidence-based echocardiographic approach to predict cardiovascular events and myocardial fibrosis in mitral valve prolapse: the STAMP algorithm. *Arch Cardiovasc Dis*. (2024) 117:173–6. 10.1016/j.acvd.2024.01.001 38368159

[52] Al-Alusi MA Lau ES Small AM Reeder C Shnitzer T Andrews CT A deep learning model to identify mitral valve prolapse from the echocardiogram. *JACC Cardiovasc Imaging*. (2025) 19:18–29. 10.1016/j.jcmg.2025.08.011 41031982 PMC12579375

[53] Ravassa S López B Treibel TA San José G Losada-Fuentenebro B Tapia L Cardiac Fibrosis in heart failure: focus on non-invasive diagnosis and emerging therapeutic strategies. *Mol Aspects Med*. (2023) 93:101194. 10.1016/j.mam.2023.101194 37384998

[54] Lin J Lopez EF Jin Y Van Remmen H Bauch T Han HC Age-related cardiac muscle sarcopenia: combining experimental and mathematical modeling to identify mechanisms. *Exp Gerontol*. (2008) 43:296–306. 10.1016/j.exger.2007.12.005 18221848 PMC2323436

[55] Hammon M Grossmann S Linz P Kopp C Dahlmann A Garlichs C 23Na magnetic resonance imaging of the lower leg of acute heart failure patients during diuretic treatment. *PLoS One*. (2015) 10:e0141336. 10.1371/journal.pone.0141336 26501774 PMC4621023

[56] Sato R Vatic M da Fonseca GWP von Haehling S. Sarcopenia and frailty in heart failure: Is there a biomarker signature? *Curr Heart Fail Rep.* (2022) 19:400–11. 10.1007/s11897-022-00575-w 36261756 PMC9653351

[57] Anagnostou D Theodorakis N Hitas C Kreouzi M Pantos I Vamvakou G Sarcopenia and cardiogeriatrics: the links between skeletal muscle decline and cardiovascular aging. *Nutrients*. (2025) 17:282. 10.3390/nu17020282 39861412 PMC11767851

[58] Attisano L Wrana JL Montalvo E Massagué J. Activation of signalling by the activin receptor complex. *Mol Cell Biol*. (1996) 16:1066–73. 10.1128/MCB.16.3.1066 8622651 PMC231089

[59] Li J Fredericks M Cannell M Wang K Sako D Maguire MC ActRIIB:alk4-fc alleviates muscle dysfunction and comorbidities in murine models of neuromuscular disorders. *J Clin Invest*. (2021) 131:e138634. 10.1172/JCI138634 33586684 PMC7880416

[60] Rutledge CA. Molecular mechanisms underlying sarcopenia in heart failure. *J Cardiovasc Aging*. (2024) 4:7. 10.20517/jca.2023.40 38455513 PMC10919908

[61] Roh JD Hobson R Chaudhari V Quintero P Yeri A Benson M Activin type II receptor signaling in cardiac aging and heart failure. *Sci Transl Med*. (2019) 11:eaau8680. 10.1126/scitranslmed.aau8680 30842316 PMC7124007

[62] Nabeel PM Kiran VR Joseph J Abhidev VV Sivaprakasam M. Local pulse wave velocity: theory, methods, advancements, and clinical applications. *IEEE Rev Biomed Eng*. (2020) 13:74–112. 10.1109/RBME.2019.2931587 31369386

[63] Armentaro G Vitale C Cassano V Magurno M Panza A Scarcelli MR Prognostic role of sarcopenia in heart failure patients. *Cardiovasc Diabetol*. (2025) 24:411. 10.1186/s12933-025-02949-5 41152875 PMC12570803

[64] Aimo A Gaggin HK Barison A Emdin M Januzzi JL. Imaging, biomarker, and clinical predictors of cardiac remodeling in heart failure with reduced ejection fraction. *JACC Heart Fail*. (2019) 7:782–94. 10.1016/j.jchf.2019.06.004 31401101

[65] Delp MD. Effects of exercise training on endothelium-dependent peripheral vascular responsiveness. *Med Sci Sports Exerc.* (1995) 27:1152–7. 10.1249/00005768-199508000-00008 7476059

[66] Dun Y Zhang W Du Y Xie K Liu Y Li C High-Intensity interval training mitigates sarcopenia and suppresses the myoblast senescence regulator EEF1E1. *J Cachexia Sarcopenia Muscle*. (2024) 15:2574–85. 10.1002/jcsm.13600 39276001 PMC11634493

[67] Karim A Muhammad T Shah I Khan J Qaisar R. A multistrain probiotic reduces sarcopenia by modulating Wnt signaling biomarkers in patients with chronic heart failure. *J Cardiol*. (2022) 80:449–55. 10.1016/j.jjcc.2022.06.006 35750555

[68] Fang H Judd RL. Adiponectin regulation and function. *Compr Physiol*. (2018) 8:1031–63. 10.1002/cphy.c170046 29978896

[69] Ybarra J Resmini E Planas F Navarro-López F Webb S Pou JM Relationship between adiponectin and left atrium size in uncomplicated obese patients: adiponectin, a link between fat and heart. *Obes Surg*. (2009) 19:1324–32. 10.1007/s11695-009-9924-5 19629601

[70] Shi K Zhang G Xu R Li XM Jiang L Gao Y Association of body composition with left ventricular remodeling and outcomes in diabetic heart failure with reduced ejection fraction: assessment of sarcopenic obesity using cardiac MRI. *Cardiovasc Diabetol*. (2025) 24:79. 10.1186/s12933-025-02639-2 39962525 PMC11834579

[71] Shi K Zhang G Fu H Li XM Yu SQ Shi R Reduced thoracic skeletal muscle size is associated with adverse outcomes in diabetes patients with heart failure and reduced ejection fraction: quantitative analysis of sarcopenia by using cardiac MRI. *Cardiovasc Diabetol*. (2024) 23:28. 10.1186/s12933-023-02109-7 38218882 PMC10787494

[72] Shinners L Grace S Smith S Stephens A Aggar C. Exploring healthcare professionals’ perceptions of artificial intelligence: piloting the Shinners Artificial Intelligence Perception tool. *Digit Health*. (2022) 8:20552076221078110. 10.1177/20552076221078110 35154807 PMC8832586

[73] Sayed N Huang Y Nguyen K Krejciova-Rajaniemi Z Grawe AP Gao T An inflammatory aging clock (iAge) based on deep learning tracks multimorbidity, immunosenescence, frailty and cardiovascular aging. *Nat Aging*. (2021) 1:598–615. 10.1038/s43587-021-00082-y 34888528 PMC8654267

[74] Calvani R Picca A Coelho-Júnior HJ Tosato M Marzetti E Landi F. Diet for the prevention and management of sarcopenia. *Metabolism*. (2023) 146:155637. 10.1016/j.metabol.2023.155637 37352971

[75] Karataş Ö Demirci S Pota K Tuna S. Assessing ChatGPT’s role in sarcopenia and nutrition: insights from a descriptive study on AI-Driven solutions. *J Clin Med.* (2025) 14:1747. 10.3390/jcm14051747 40095876 PMC11900272

